# Coverage, Opportunity, and Challenges of Expanded Program on Immunization among 12–23-Month-Old Children in Woldia Town, Northeast Ethiopia, 2018

**DOI:** 10.1155/2019/5302307

**Published:** 2019-12-31

**Authors:** Ayele Mamo Abebe, Mesfin Wudu Kassaw, Alemu Birara Zemariam, Nathan Estifanos Shewangashaw

**Affiliations:** ^1^Department of Nursing, Debre Birhan Health Sciences College, Debre Birhan 37, Amhara, Ethiopia; ^2^Department of Nursing, College of Health Sciences, Woldia University, Amhara, Ethiopia; ^3^Department of Nursing, College of Health Sciences, Wollo University, Amhara, Ethiopia

## Abstract

**Background:**

Each year, immunization averts an estimated 2–3 million deaths from diphtheria, tetanus, pertussis, and measles. In 2011, nearly 107 million infants (83%) worldwide received at least 3 doses of DTP vaccine; however, approximately 22.4 million failed to receive 3 doses, and this causes large numbers of children susceptible to vaccine-preventable diseases and death. Nearly 8.4 million received at least 1 DTP dose. The aim of this was to assess coverage, opportunity, and challenges of EPI among children of age 12–23 months in Woldia town, Amhara region, Northeast Ethiopia.

**Method:**

In this mixed community-based cross-sectional study, 389 study subjects were selected by using a multistage sampling method. Interviewer-administered structured questioners were used. Data were entered and analyzed using SPSS Version 20 and presented by using tables and figures. Documented/recorded file from the qualitative data were transcribed into word narrative. Finally, it was presented by thematic analysis.

**Result:**

A total of 389 mothers/caretakers were interviewed. Based on vaccination card and mothers/caretakers' recall, 385 (99%) of the children took at least a single dose of vaccine. From total children, 4 (1%) were not immunized at all, 44 (11.3%) were partially immunized, and 343 (87.7%) were fully immunized. The dropout rate was 9% for BCG to measles, 2.4% for Penta1 to Penta3, 8.3% for penta1 to measles, and 1.6% for pcv1 to Pcv3. A qualitative study revealed that workload, shortage of vaccine, and noncompliance of the mother/caretaker for the next scheduled date was the major challenge faced by health professionals and health extension workers.

**Conclusion:**

Vaccination coverage was low compared with the Millennium Development Goals target. It is important to increase and maintain the immunization level to the intended target. Thus, the town health office and concerned stakeholders need to work more to improve the performance of the expanded program on immunization in this area.

## 1. Introduction

Expanded Program on Immunization (EPI) was begun by the World Health Organization (WHO) in 1974 [1, 2]. About 107 million babies (83%) globally had got at least 3 doses of DTP vaccine; but, nearly 22.4 million miscarried to acquire 3 dosages, exposing huge numbers of children susceptible to vaccine-preventable diseases and death [3, 4]. One out of five infants worldwide does not receive 3 life-saving doses of diphtheria, tetanus, and pertussis vaccine [5].

In sub-Saharan Africa, 4.4 million children died yearly due to transmittable diseases that could be avoidable by immunization [6, 7]. The occurrence of vaccine-preventable diseases are associated with poor immunization coverage and challenges and setup were not fully equipped in sub-Saharan African countries [8].

EPI was begun in 1980 in Ethiopia with the purpose of reducing decease and disease of children and mothers from vaccine-avoidable diseases [9]. To achieve maximal protection against vaccine-preventable diseases, a child should receive all vaccines [10–13]. It has been also recognized that vaccine-preventable diseases are responsible for 16% of under-five mortality in Ethiopia [14].

Eventhough the Ethiopian government did different efforts, the coverage rates stayed highly small for several centuries [16, 17]. As explained by EDHS 2016, two from five children aged 12–23 months (39%) had got totally fundamental immunizations at some time, and also 22% have gained vaccines at a suitable period of time. There was an increment of fully vaccinated children aged 12–23 months with a percentage from 24% to 39% [18]. Based on the EDHS 2016 report, there is a wide difference among regions concerning full immunization coverage ranging from 89% in Addis Ababa to 15% in the Afar region due to different challenges and opportunities as explained in other studies [16–20].

But, in Woldia town there was no previous study that shows the coverage and dropout rate of EPI and asseses different challenges and opportunities of EPI. Therefore, the aim of this study is to determine the level of immunization coverage and dropout rate and to explore challenges and the opportunity of EPI among all pairs of mother to children aged 12–23 months in Woldia town.

## 2. Methods and Materials

### 2.1. Study Area and Period

A community-based cross-sectional study using both quantitative and qualitative methods was conducted in Woldia town. Woldia is the capital of the North Wollo Zone which is located at about 360 km from Bahir Dar capital city of Amhara region and 520 km away from Addis Ababa capital city of Ethiopia; this town has an elevation of 2112 m above sea level which is administratively structured by ten Keble, and according to 2007 Ethiopia national census, the projected population size of the town is 79,667 in 2010 of whom 40,303 are men, 39,365 are women, and 2250 are children of age 12–23 months with a total household of 16347. The majority of the inhabitants practiced Ethiopian orthodox with 80.49% while 18.46% of the population are Muslim. Two largest ethnic groups reported in this were the Amhara (93.92%) and Tigray (4.32%) [28] reasons provided by other caregivers on other similars. Woldia has 1 general hospital, 2 health centers, four health posts, and more than nine private clinics. Vaccination service is being provided in 2 health centers and hospitals free of charge. The study was conducted from April 26–May 11, 2018. Mothers or caretakers aged 18 years and above with children aged between 12 and 23 months were included in this study.

### 2.2. Sample Size Calculation

The sample size for this study was calculated using the single population proportion formula based on the following assumption. The sample size calculated by using single population proportion formula by assuming P = 76% was taken from the Gondar study (22), and design effect = 1.5. Design effect is

(1) $$\begin{array}{c} n=\frac{z_{\alpha /2}^{2}p\left(1-p\right)}{w^{2}}, \end{array}$$

where *n* is sample size, *Z*_*α*/2_: with 95% confidence interval equal to 1.96, *p*: estimation of EPI coverage which is 76%, *w*: margin of error which is 1−confidence level = 1–0.95 = 0.05, *n* = (1.96)^2^ *∗* (0.76) (1 − 0.76)/(0.05)^2^ = 280 *∗* 1.5 = 420.

Our total population is < 10,000, so we use the following correction formula:

(2) $$\begin{array}{c} n_{f}=\frac{\text{no}}{\left(N+\text{no}/N\right)},\\ n_{f}=\frac{\text{no}}{\left(1+\text{no}/N\right)},\\ n_{f}=\frac{420}{1+420/2250}=354. \end{array}$$

By adding 10% nonresponse rate, we get a final sample size of 389, where, *n*_*f*_ = Final sample size, no = total sample size from the abovementioned formula, and *N* = total population (children aged 12–23 months in Woldia town in 2010).

### 2.3. Sampling Technique

**Figure 1:**
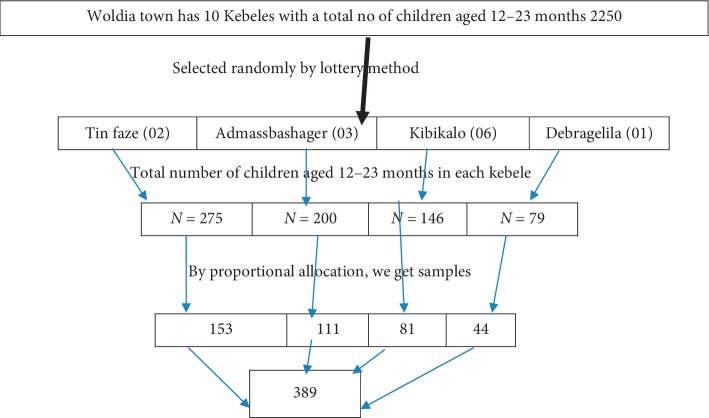
Schematic presentation of the sampling procedure.

Multistage sampling was used to take the appropriate sample. Initially, Woldia town had 10 kebeles and out of those 4 kebeles were selected (40% of the kebeles will consider to be included in the study) by the simple random technique (lottery method). The total sample size was allocated proportionally to each kebeles depending on the total number of children aged 12 to 23 months. The sampling frame was obtained from health extension workers' registration books. In each kebeles, the first household was selected randomly from the central location of the kebeles. The subsequent household was selected according to the inclusion criteria based on the principle of the next nearest household. Households in the kebeles were visited until the proportionally allocated sample size for each kebeles was fulfilled. One child was selected randomly from those households having two and more children. The sample for an in-depth interview was selected by the nonprobability purposive sampling method. A total of 11 in-depth interviews were conducted among health professionals and health extension workers (HEW) who work under an expanded program of immunization (EPI) in Woldia town (Figure 1).

### 2.4. Operational Definition

The following operational definitions were used:

  Fully vaccinated: children are considered as fully vaccinated when they have received a vaccination against tuberculosis (BCG), three doses each of the Penta and PCV, and four doses of polio vaccines and measles vaccination by the age of 12 months (19, 20).
  Partially vaccinated: children are considered as partially vaccinated when they miss at least one dose of the abovementioned vaccines on a fully vaccinated definition (19, 20).
  Unvaccinated: children are considered as unvaccinated when they did not receive any dose of the abovementioned vaccines on the fully vaccinated definition (19, 20).
  Vaccinated: children are considered as vaccinated when they took at least one dose of the abovementioned vaccines on fully vaccinated definition (19, 20).
  Dropout rate: this is the rate difference between the first and the last dose or the rate difference between the initial vaccine and the last vaccine (19, 20).
  Health service utilization of mothers: utilization of ANC, contraceptive, and delivery service by the mothers from government or private health institutions (19, 20).
  Coverage by card only: coverage by card only meant coverage calculated with numerator based only on the documented dose, excluding the numerator of those vaccinated by history.
  Coverage by card plus history: coverage by card plus history meant coverage calculated with numerator based on card and the mother's report (19, 20).
  Caretaker: caretaker is the most responsible person who provides care for the child who has no mother due to different reasons (death, separated from husband, and others) (19, 20).
  Kebele: it is the smallest administrative unit in Ethiopia. It is part of a woreda (district), itself usually part of a zone. Each kebele consists of at least five hundred families or the equivalent of 3500 to 4000 persons.

### 2.5. Data Collection Tool and Procedure

The questionnaire was adapted and modified from the Ethiopian EPI Survey of 2012, DHS, and other previous study questionnaires. The research aimed to collect both quantitative and qualitative data. For the cross-sectional survey, interviewer-administered questionnaires were administered to mothers or caretakers. It was translated into Amharic and translated back to English by professional translators for consistency. The child's vaccination dates a number of doses, and dates of other visits to the health facility was extracted and collected from the vaccination card and history. Information about the mother's knowledge of vaccination and the program and accessibility to the nearest health facility was obtained through verbal information. If the vaccination card was unavailable for the child, the mothers/caretakers were asked for vaccination history. The number of doses the child took and how (the route of vaccine administered) were used to identify the given antigen for the child. Respondents who were not available during the first visit were revisited within the day or during the interviewer's stay in the area. For data collectors, a two-day intensive training was given before data collection. The training was given in the Amharic language on how to ask and fill the question and selection criteria of households and children and how to approach the mothers/caretakers.

An in-depth interview schedule was used for collecting qualitative data. The schedule contained an open-ended question about challenge and opportunity of EPI. The interviews were documented appropriately by participant words, and the participants speaking were recorded by tape. Finally, their speaking was transcribed to English words. Data collection was performed from April 26–May 11 for two weeks. Before the actual data collection days, the questionnaire was pretested for completeness and appropriateness to the local context on 5% (20 mothers/caretaker) of a mother with children aged 12–23 months in Defergie Keble and was modified accordingly.

### 2.6. Data Quality and Control Measure

The questionnaire was prepared in English language and translated to Amharic language and again translated to English by professional translators to increase its quality and ensure that the translated version did not alter the meaning of the questionnaire. The pretest was done in 5% of the sample size at a similar setting near in the study setting (defergie Keble) one week before scheduled data collection day to improve the tool. Based on the pretest, the necessary modification was performed. The data collectors were trained for two days on principle, ethical considerations, and how they collected the data, and strict supervision of the data collector was performed. Questionnaires were checked for completeness at the end of each day of data collection by the principal investigator.

### 2.7. Data Processing and Analysis

Quantitative data were coded, entered, and analyzed using SPSS version 20 for Windows. Summary statistics such as percentages, frequency, and graphical techniques were used.

A recorded or paper documented file from the qualitative data was transcribed into Microsoft word 2010. Transcripts were read and understood by all participants. Similar topics were grouped together, and those with common features were clustered together until the final themes and subthemes emerged. Themes and their subthemes were arranged based on their commonality.

### 2.8. Ethical Consideration

Ethical clearance was obtained from the Faculty of Health Science, Department of Nursing, Woldia University. An official letter was written by the Department of Nursing to Woldia city of Administration to get permission and support to each respected kebeles. After a brief explanation of the objectives and purpose of the study, verbal informed consent was obtained from each study participant. Participants were also informed that participation was on a voluntary basis and they have the right to stop their participation at any time. Study participants were also informed that all data obtained from them would be kept confidential by using codes instead of any personal identifiers.

## 3. Result

### 3.1. Sociodemographic Characteristics of the Study Population

A total of 389 mothers/caretakers of children aged 12–23 months were interviewed with a response rate of 100%. The majority, 195 (50.1%), were within the age of 25, 31, 88 (22.5%) were between 32–38 years, 71 (18.3%) were between 18–24 years, and the remaining 35 (9%) were 39 and above with a mean age of 29.59 and median of 28, ranging from 19–44 years (Table 1). The immediate caregivers of the children were mothers (97.9%), fathers (1.8%), and other family members (0.3%). Concerning marital status, 84.8% of the caregivers were currently married followed by 11.6% divorced, and the rest 3.6% were widowed. With regard to religion, 324 (83.3%) were orthodox while 56 (14.4%) were Muslim, and rest 9(2.3%) were protestant. The majority of 371 (95.4%) belong to the Amhara ethnic group. Among the interviewed caregivers, 30.3% had elementary level knowledge, 54.7% had secondary level knowledge and above, 4.1% were able to read and write, and the rest 10.8% were unable to read and write. By occupation, 226 (58.1%) were housewives and 52 (13.4%) were government employees. With regard to the income of respondents, 66 (17%) were with monthly income less than 500 birrs and 258 (66.3%) were with monthly income greater than 500 birrs (Table 1).

### 3.2. Family Size and Characteristics of the Child

Among the respondents 171 (44%) have one child, 161 (41.4%) have 2 or 3 children, 50 (12.9%) have 4 or 5 children, 7 (1.8%) have more than 6 children. The average family size of the study population was 4 ranging from 2 to 9, in which most families had less than 5 members (70.4%). The mean age of the children was 17.38 months (range, 12–23), and 218 (56%) were of the male gender (Table 2).

### 3.3. Immunization Coverage of Children Aged 12–23 Months

Only 98 (25.2%) of mothers/caretakers showed the child vaccination card during the survey. From a total of 389 children aged 12–23 months selected and included in this study, 385 (99%) of them have taken one or more of the recommended vaccines and 4 (1%) were unvaccinated according to finding from card plus history. Of the total vaccinated children, 341 (87.7%) of them finished all the recommended doses and 44 (11.3%) did not complete the entire doses.

Out of the total surveyed children aged 12–23 months, vaccination card was only seen and confirmed for 98 (25.2%) children. Coverage by card only was calculated by taking children who had a vaccination card as a numerator. From 98 vaccinated by card only, 98.9% received OPV1 followed by BCG (92.8%) and OPV2 (96.9%). Penta 3 was taken by 86.7%, and the measles vaccine was taken by 72.4%.

Based on the vaccination card and the mother's/caretaker's recall, 385 (99%) of the children took at least a single dose of vaccine. From the total study participants, 341 (87.7%) were claimed fully-immunized, 44 (11.3%) were partially vaccinated, and 4(1%) were unvaccinated (Table 3).

### 3.4. Dropout Rate for Vaccines and Reasons for Defaulting from Vaccination Service

The problem of the dropout was with the subsequent antigens specifically measles which are given as last vaccines to end the entire immunization programme. There was an increase, according to the findings from the field survey in the number of children who defaulted on the vaccines from DPT1 and OPV1 to Measles. Forty-two children (10.8%) defaulted for measles getting from both recall and card. The DPT-HepB-Hib1-measle dropout rate for children was 8.3% and DPT-HepB-Hib1-DPT-HepB-Hib3 and the pcv1-pcv3 dropout rates were 2.4% and 1.6%, respectively. The overall dropout rate (from BCG-measles) was 9%.

The findings of the survey showed that 2.3% of caregivers reported that the reason for not completing child vaccination was the lack of awareness about completing vaccination schedule and same 2.3% not knowing whether to come back for second and third vaccination, 2.1% reported fear of side effect, 1.8% reported vaccination time is inconvenient, and same 1.8% because of no vaccination at health facility at the time of vaccination day. There were 4 children who were never vaccinated and different reasons were given by mothers, and the reason given by more than half for not vaccinating their children was lack of awareness on importance of vaccination and the remaining respondents answered fear of side effect and the child was sick as a reason (Table 4).

### 3.5. In-Depth Interview Findings

#### 3.5.1. Theme 1: Challenges Faced by HW and HEW

Health extension workers and health professionals highlighted that they faced challenges when implementing EPI, those with shortages of vaccines and workload.

*Subtheme 1.1: Shortage of Vaccines*. In the health facility, professionals sometimes experience shortages of vaccines which make it difficult for them to follow-up on children who missed their inoculations. The challenges are the shortage of vaccines; sometimes, we find that at 10 weeks, the mothers come and do not find the vaccines. There are some vaccines like rotavirus that cannot be given after 6 months; thus, children miss some of the doses. This was confirmed by a participant who said,

  You know, there are a lot of problems that we are facing related to the implementation of EPI which include the fact that those vaccines are not enough or are not available to us to immunize the children who come here expecting us to immunize them

*Subtheme 1.2: Workload*. Participants reported that workload reduces the accuracy of records made in the course of implementation and reduces the chances of counseling clients on the importance of vaccines, and it causes clients to wait in the queue for long leading to drop out rates. Many clients are reluctant to return for subsequent visits if they experienced long waiting times in prior visits. The participant said that the cause of workload is inadequate workspace and high patient/staff ratio. This quote from a participant explains the effect of inadequate workspace. “You can realize that you might want to divide tasks, and built becomes difficult because of small working place”. Small working space results in crowding of patients, which affects privacy of clients and quality of services given.

*Subtheme 1.3: Noncompliance of Mothers with Scheduled Return Dates*. The study revealed that all mothers did not comply with the instructions given by nurses during EPI implementation. This finding was confirmed by a participant who said, “It feels nice as a nurse to do immunizations if mothers respect the return dates, and it is discouraging when mothers sometimes do not bring the children on the recommended dates because this seems like we are not doing our work, emphasizing that mothers must respect their return dates.”

Noncompliance might result from a poor understanding of immunization by mothers of children who are supposed to be immunized and loss of vaccination card which remembers the day of return for the next schedule. It is claimed that most mothers do not know which diseases are prevented by which vaccines or how many doses of each are needed, and that is the reason why they sometimes miss the scheduled dates.

#### 3.5.2. Theme 2: Possible Solutions to Relieve Faced Challenges

A participant was put different solutions in order to alleviate the above challenges. Among those,

- Provide more workspace
- Adequate supply of the vaccine
- Employing more staff
- Intensive health education about when to return, proper use of the card, and what vaccine was given at what age of the child and what type of disease is prevented by that specific vaccine

#### 3.5.3. Theme 3: Opportunity That Increases EPI Coverage

*Subtheme 3.1: Health Professional's Awareness Regarding the Internal Referral System*. This was confirmed by a participant who said, “Most health professionals are aware of the internal referral system. They check the immunization status of children who come for other child health services and refer to the immunization room when necessary. Most health professionals also advise mothers who come for maternity services to vaccinate their child.”

*Subtheme 3.2: Information Accessibility*. It was confirmed by a participant who said “our community gets information regarding immunization easily from media, HEW, and other sources since it is the urban community,” so if the community has an awareness regarding the benefit, session needed to complete and the age to start and finish vaccination increases the utilization of EPI.

*Subtheme 3.3: Near Health Facility*. As we know, the factor that affects vaccination of children is a distance to health institutions and accessibility of transport. The participant said, “in Woldia Town, almost all mothers get immunization service at a near health facility, easily accessible of transport, so this favors the increment of taking vaccination and completing the schedules.”

*Subtheme 3.4: Outreach Service Delivery*. HEW said that all kebeles have health an extension worker, so the HEW always search unvaccinated and partially vaccinated children and after getting those children, they give health education about vaccine advantages for the mother and give appointment for the mother for the next schedule to go to the nearest health care facility.

## 4. Discussion

This study was conducted in the urban community to assess coverage, opportunity, and challenges of EPI among children aged 12–23 months residing in selected kebeles of Woldia town found in North Wollo Zone, Amhara regional state of Ethiopia.

Immunization coverage was assessed using the availability of vaccination cards and maternal recall (history). Based on immunization card and history, 343 (87.7%) children were fully vaccinated, and 44 (11.3%) and 4 (1%) were partially vaccinated and unvaccinated, respectively. According to the EPI schedule of Ethiopia, OPV and pentavalent vaccines are being given with similar schedule; however, OPV coverage was slightly higher than pentavalent which could be due to the fact that there are national immunization supplement campaigns for OPV and measles. The result showed increased vaccination coverage when compared with the previous study done in different areas [20–23]. This may be due to increasing access to vaccination and community awareness from time to time. But, this study is lower than the study conducted in Debre Markos town in 2016, which showed a total number of fully vaccinated children aged 12–23 months was 91.7% [24]. This may be due to no vaccine available at the time of vaccination in the study area.

The overall dropout rate for this study was 9%. The dropout rate from pentavalent1 to measles (8.3%) was higher than the dropout rate from pentavalent1 to pentavalent3 (2.4%) and from PCV1 to PCV3 (1.6%). Because of the relatively long interval between the third dose of pentavalent and measles, a number of children may not return for the measles vaccine. This may have made mothers forget vaccination appointment dates, and this makes the coverage rate for this antigen to be lower than other vaccines. However, this finding is a lower dropout rate compared with other studies such as EDHS 2011, which showed the DPT and polio dropout rate were 43% and 46%, respectively, and a study done Yirgalem town in Sidama zone showed the penta1 to measles dropout rate was 18% [15, 20]. The possible explanation could be that EDHS 2011 has used samples from all the regions of Ethiopia including developing regions Afar, Somali, Benishangule Gumuz, and Gambella. The residents of these regions are pastoral who may have low awareness and health-seeking behavior including vaccination of their children. The people who live in this region may have less educational status and may live far from the health-care institutions. But still, our study has a higher dropout rate from a study conducted in Debre Markos town, Amhara region, which showed the overall dropout rate was 5% [24]. This is due to the fact that there are different challenges in our study area like workload and no vaccination on the day of vaccination.

This study showed that there is decline in coverage of immunization from BCG (96.9%) to measles (88.2%) and the proportion of fully immunized children (87.7%) which indicated that there was significant proportion of defaulting children.

The mother or care givers mention different reasons for not completing immunization to children. These reasons were, 2.3% of caregivers reported that the reason for not completing child vaccination was lack of awareness about completing vaccination schedule and same 2.3% not knowing whether to come back for the second and third vaccination, 2.1% reported fear of side effect, 1.8% reported vaccination time is inconvenient, and same 1.8% because of no vaccination at health facility at the time of vaccination day. Most of the reasons given by the care givers have similarity with the reasons provided by other caregivers on other similar studies [20, 25, 26, 27].

From the qualitative study, workload due to staff shortage and inadequate workspace, shortage of vaccine, and noncompliance of a mother for the next scheduled date were the major challenges faced by a health professional and health extension worker. This is almost similar to a study conducted in Arebegona district, Southern Ethiopia [24].

## 5. Strength and Limitation of the Study

### 5.1. Strength

A qualitative method was included to answer and clarify some issues on the study.

Only children aged between 12 and 23 months were included in the study which shows recent vaccination program performance.

### 5.2. Limitation

The study is conducted only in the town, which does not represent the great majority of the rural community around Woldia town.

The study participants may create social desirable bias during the interview.

## 6. Conclusion and Recommendation

The level of immunization coverage was found to be low among children aged 12–23 months in Woldia town compared with the national MDG target (at least 90.0%) to be achieved by 2015. Reasons for incompletion are mostly because of the lack of awareness about completing vaccination schedule not knowing whether to come back for the second time and fear of side effects. Also, as a reason for not vaccinating their child, most respondents replied that lack of awareness on the importance of vaccination and the remaining respondents answered fear of side effect and the child was sick as a reason.

Workload, noncompliance of a mother for next schedule vaccination date, and shortage of vaccine was the major challenges faced by health professionals and health extension workers. Near the health facility, information accessibility regarding immunization and health worker awareness regarding the internal referral system was a good opportunity that increases immunization coverage.

## Figures and Tables

**Table 1:** Sociodemographic characteristics of the caregiver in Woldia town, North Wollo Zone, Northeast Ethiopia, May 2018 (*n* = 389).

	Frequency	Percent (%)
Age		
18–24 years	71	18.3
25–31 years	195	50.1
32–38 years	88	22.5
>_39 years	35	9

Educational level		
Unable to read and write	42	10.8
Able to read and write	16	4.1
Elementary	118	30.3
Secondary and above	213	54.7

Marital status		
Married	330	84.8
Widowed	14	3.6
Divorced	45	11.6

Occupation	Frequency	Percent (%)
House wife	226	58.1
Farmer	27	6.9
Government employee	52	13.4
Merchant	51	13.1
Daily laborer	9	2.3
Other	24	6.2

Religion		
Orthodox	324	83.3
Muslim	56	14.4
Protestant	9	2.3

Ethnicity		
Amhara	371	95.4
Tigray	10	2.6
Oromo	5	1.3
Other	3	0.8

Monthly income		
<500	66	17
>500	258	66.3
Unknown	65	16.7

**Table 2:** Family size and characteristics of a child in Woldia town, Northeast Ethiopia, May 2018 (*n* = 389).

	Frequency	Percent
Sex of child		
Male	218	56
Female	171	44

Age of child		
12–15 months	161	41.3
16–19 months	83	21.4
20–23 months	145	37.2

Number of family size		
2–4	274	70.4
5–7	106	27.2
>7	9	2.3

Children from mother		
1	171	44
2–3	161	41.4
4–5	50	12.9
>_6	7	1.8

**Table 3:** Vaccination coverage of children aged 12–23 months based on child vaccination card and the mother's recall in Woldia town, North Wollo Zone, Northeast Ethiopia, 2018 (*n* = 389).

Vaccines	Coverage by card		Coverage by recall		Coverage both by card and recall	
	Frequency	%	Frequency	%	Frequency	%
BCG	91	23.4	286	73.5	377	96.9
OPV0	92	23.7	286	73.5	378	97.2
OPV1	97	24.9	285	73.3	382	98.2
OPV2	95	24.4	284	73	379	97.4
OPV3	86	22.1	282	72.5	368	94.6
Penta1	89	22.9	285	73.3	374	96.2
Penta2	93	23.9	284	73	377	96.9
Penta3	85	21.9	280	72	365	93.9
Pcv1	89	22.9	284	73	373	95.9
Pcv2	95	24.4	284	73	379	97.4
Pcv3	86	22.1	281	72.2	367	94.3
Rota1	91	23.4	284	73	375	96.4
Rota2	88	22.6	281	72.2	369	94.8
Measles	71	18.3	272	69.9	343	88.2

**Table 4:** Reasons given by caregivers for not completing children vaccination, Woldia Town, Northeast Ethiopia, May 2018 (*n* = 389).

Reason of defaulting vaccination	Frequency	Percent
Vaccination site far away	2	0.5
Child was sick	1	0.3
Vaccination time is inconvenient	7	1.8
Lack of awareness about completing vaccination schedule	9	2.3
Not knowing vaccination time and site	1	0.3
Not knowing whether to return back for second or third visit	9	2.3
Fear of side effect	8	2.1
No vaccine at health facility	7	1.8

## Data Availability

Data supporting the conclusions of this article are available by request to Ayele Mamo. The relevant raw data will be made available to researchers wishing to use them for noncommercial purposes. The Supplementary data will be put below the acknowledgement.

## Abbreviations

BCG: Bacillus Calmette-Gurin
DHS: Demographic health survey
DPT: Diphtheria, Pertussis, Tetanus
EC: Ethiopian calendar
EDHS: Ethiopian Demographic and Health survey
EPI: Expanded Program on Immunization
GVAP: Global vaccine action plan
HEB: Hepatitis B
MCV: Measles containing Vaccine
MDG: Millennium development goal
MOH: Ministry of health
OPV: Oral Polio Vaccine
PCV: Pneumococcal Conjugate Vaccine
SNNPR: Southern nation nationality people region
SPSS: Statistical package for social science
UNICEF: United Nations International Children's Emergency Fund
VPD: Vaccine preventable disease
WHO: World Health Organization.
